# Editorial: Psychosomatic medicine in general hospitals: Cross-Disorder and interdisciplinary collaboration

**DOI:** 10.3389/fpsyt.2022.1099678

**Published:** 2022-12-13

**Authors:** Wenhao Jiang, Yuqun Zhang, Jessica A. Turner, Yonggui Yuan

**Affiliations:** ^1^Department of Psychosomatics and Psychiatry, School of Medicine, ZhongDa Hospital, Southeast University, Nanjing, China; ^2^School of Nursing, Nanjing University of Chinese Medicine, Nanjing, China; ^3^Department of Psychiatry and Behavioral Health, Wexner Medical Center, The Ohio State University, Columbus, OH, United States

**Keywords:** psychosomatic medicine, Consultation-Liaison Psychiatry, general hospital, interdisciplinary, psychosomatic disorder

Psychosomatic medicine focus on the etiology, diagnosis, treatment, and prevention of psychosomatic disorders (1). Although the definitions of psychosomatic medicine may vary, the burden of psychosomatic disorders has been well-recognized. During the past decades, increasing efforts have been made to improve our understanding of psychosomatic disorders and related risks. The management of these disorders is critical in general hospitals. Identifying psychosomatic disorders, accurate early diagnosis, treatment, and conducting clinical research have received increasing attention from physicians of all departments (2).

We initiated this special issue to encourage interdisciplinary studies in this field. This collection presented 23 manuscripts, including original research, reviews, and case reports worldwide. In addition, the Chinese Society of Psychosomatic Medicine (CSPM) has broadcasted this Research Topic to the local community. We covered papers introducing cross-cultural psychosomatic models and concepts, case reports, the development of assessment tools, related psychosomatic issues in psychiatric disorders, and psychosomatic problems in healthcare workers.

## Cross cultural psychosomatic models and concepts

Psychosomatic medicine models, concepts, and current clinical practice vary in regions. A little more than half a century ago, European countries started to play significant roles in this field. Sadlonova et al. introduced the development of multimodal psychosomatic treatment in Germany. They investigated treatment outcomes and long-term predictors among inpatients with such treatment programs. The authors discovered medium to considerable improvements in psychological and physical symptoms and the quality of life. They also found better quality of life improvement, and antidepressants during hospitalization might lead to a better prognosis. This finding emphasized the significance and effectiveness of multimodal psychosomatic treatment for inpatients.

Zerbinati et al. discuss a multicenter study focusing Consultation-Liaison Psychiatry (CLP) practice in Italian general hospitals. The authors compared CLP visits data of 3,943 patients from 10 Italian hospitals during 2018 with 4,183 participants and their data in 1998. Over 20 years, an increased CLP proportion was detected in surgical and onco-hematologic departments. Depressive disorders remained the most frequent diagnosis. The author stated that the significantly increased CLP workload required better organization and autonomy in Italian CLP services.

Bai et al. researched the history of depression as a concept in China. They analyzed materials, including newspaper stories, medication advertisements, and medical texts. They believed that the depression evolved from a hazy and ambiguous concept into a specific disease in the late Qing Dynasty and early Republican. This progress might parallel western medicine entering the Chinese pharmaceutical market in the 1920s. In addition, the authors explored the stereotypes related to depression during the early days. Bai's et al. paper is an exciting exploration of the history of psychosomatic medicine in China.

## Psychosomatic symptom assessments in general hospitals

The assessment tools of psychosomatic symptoms, risk factor analysis, and interaction between them are essential in collecting clinical information for further investigations. Zhang J. et al. investigated sex, age, depression, insomnia, psychological stress, resilience, and perceived social support among patients with medically unexplained symptoms (MUS) in a general hospital's psychological clinic. They suggested middle-aged female patients were at high risk of presenting MUS and thus needed a comprehensive evaluation and timely intervention.

Cao et al. explored the accuracy of several assessment tools in detecting DSM-5 somatic symptom disorder in a general hospital. They found that Somatic Symptom Disorder—B Criteria Scale 12 or Whiteley-8 alone might show a better ability for detecting somatic symptom disorder. Li et al. validated the Chinese version of the Somatic Symptom Scale-8 (SSS-8) in outpatients. The authors found that this might be a reliable tool for assessing somatic symptoms in research and clinics.

A network approach was used by Wan et al. to explore the association between depressive symptoms in type 2 diabetes mellitus. The authors collected information from the electronic health record of 52,139 patients through deep learning. Their study's learning model and network alterations could effectively identify depressive symptoms.

Guo et al. explored the relationship between personality traits, alexithymia, and microbiome in patients with functional gastroenteropathy and generalized anxiety disorder. The authors found that the comorbidity related to the abundance of Fusobacterium. Functional gastroenteropathy alone might be related to the abundance of Haemophilus. Specific traits such as affective recognition and expression disorder, neuroticism, and negative cognition might be related to the abundance of Fusobacterium and Megamonas.

## Psychosomatic issues in psychiatric disorders

This collection also included diverse research regarding psychosomatic issues in psychiatric disorders. In anxiety disorders, Hong et al. examined the interaction between rs2071345 and alcohol dependence behavior on anxiety symptoms of male problem drinkers. The authors suggested that rs2071345 was playing a role in this specific group. On the other hand, Chu et al. examined the BDNF Val66Met polymorphism, Plasma BDNF level, and anxiety traits in panic disorder. They found that Met/Met genotype might be more likely to exhibit risky anxiety traits. Yang et al. studied the association between subclinical hypothyroidism and anxiety in depressive patients. This was the first episodic and drug-naïve group, and the authors found that serum thyroid stimulating hormone level may be a promising biomarker of anxiety symptoms among them. The research by Shen et al. also focused on biomarkers for anxiety. Serum S100B and cytokines (IL-1β, IL-2, IL-4, and IL-10) were examined in untreated generalized anxiety disorder cases and healthy controls. They found that the combination of S100B and cytokines had a better diagnosis value with high accuracy.

Major depressive disorder (MDD) and related issues also received much attention. Zhou H. et al. studied the negative emotional bias in depressive patients. The anterior cingulate cortex and right insula were found to mediate the interoception dysfunction and negative emotional bias of MDD. The study from Zhou Y. et al. also focused on the brain function of MDD. However, they studied non-suicidal self-injury behaviors, the amplitude of low-frequency fluctuation, and regional homogeneity. The authors suggested that the default mode network and visual network might be related to such behavior. Wu et al. reviewed clinical studies of MDD using the Delphi method, and they suggested this method can be helpful both in clinical and research settings.

Bipolar disorder and schizophrenia were also covered. Wang et al. studied the effectiveness of hypomania checklist-32 in screening bipolar disorder while considering personality traits' influence. They believed that higher typical extraversion and neuroticism might lead to higher hypomania checklist-32 score. Clinicians should pay attention to these false positives while using this tool. Cheng et al. focused on the relationship between suicidal risk and childhood maltreatment in schizophrenia patients. Among all the risks, they found that schizophrenia with more positive symptoms relapses had more childhood trauma, stress, and suicidal risk.

Chen et al. investigated the impulsive personality traits and negative mood states in bulimia. The study included 146 female bulimia patients and identified three linked clusters (“ED-specific symptoms,” “impulsivity,” “anxiety,” and “depression”). The authors showed that the cognition of “shape dissatisfaction” played a key role, and impulsivity and emotional symptoms contributed to the development of this eating disorder.

## Care for healthcare workers

Two studies focused on psychosomatic health in healthcare workers, especially nurses. Huang et al. investigated a specific group of the nurse in the neonatal department. They explored the lifestyle and social factors contributing to mood disorders and functional dyspepsia. Nearly half of the participants exhibited mood symptoms such as depression and anxiety. Most neonatal nurses suffering from mood disorders also presented functional dyspepsia. Poor sleep and smoking might be risk factors among them. Yin et al. investigated nurses from psychiatric departments. They studied the relationship between coping style, sleep, and burnout. The authors suggested that coping style mediated between sleep quality and burnout. They called for developing coping skills to balance work and life in this group.

## Case reports

Finally, several case reports were presented in the collection, and these cases described how physicians recognized and treated patients exhibiting psychosomatic symptoms. Lipkes et al. described a woman diagnosed with Hashimoto's thyroiditis. She was brought to the psychiatric emergency department with auditory hallucinations and persecutory delusions for the first time. The patient was treated effectively with IV thyroid replacement and antipsychotics. The authors emphasized the importance of a thorough medical workup for new-onset psychosis and called for future consensus in treatment choice.

Zhang Y. et al. described a woman who suffered from an unhealed sore and ulcer of a surgical wound after 10 years diagnosed with breast cancer. Combined with her mood symptoms, the patient's condition was identified as Qi (or vitality) deficiency in the view of Chinese traditional medicine. Herb medicine and a regimen based on Internal Vitality were given to her. The authors observed healed sore and ulcers over 6 months.

Dong et al. reported that a woman suffered from significant limb edema for 2 months. She was previously diagnosed with pituitary adenoma and received treatments. The authors believed the edema was caused by tumor recurrence, but all examinations provided negative results. Her existing severe mood problems then came into sight, and she was diagnosed with major depressive disorder after CLP. The patient's limb edema dramatically subsided after Deanxit and Tandospirone treatment.

To sum up, we expect that this special issue will expand knowledge in psychosomatic medicine and collaborative research. These studies will help reveal cross-cultural issues and underrepresented populations worldwide. In addition, the CSPM will continuously push forward domestic psychosomatic research and international collaborations. Finally, we hope the research achievement in biopsychosocial mechanisms will contribute to clinical practice.

## Author contributions

WJ has prepared the first draft of this editorial. YZ, JT, and YY have revised the first draft and contributed to the final version of the manuscript. All authors approved the submitted version.
